# Special Considerations in Pediatric Endoscopic Skull Base Surgery

**DOI:** 10.3390/jcm13071924

**Published:** 2024-03-26

**Authors:** Bastien A. Valencia-Sanchez, Jeeho D. Kim, Sheng Zhou, Sonja Chen, Michael L. Levy, Christopher Roxbury, Vijay A. Patel, Sean P. Polster

**Affiliations:** 1 School of Medicine and Health Sciences TecSalud, Tecnologico de Monterrey, Monterrey 64710, Mexico; 2 Department of Otolaryngology-Head and Neck Surgery, Naval Medical Center San Diego, San Diego, CA 92134, USA; 3 USC Caruso Department of Otolaryngology-Head and Neck Surgery, Los Angeles, CA 90033, USA; 4 Department of Neurosurgery, University of Chicago, Chicago, IL 60637, USAspolster@uchicago.edu (S.P.P.); 5 Division of Pediatric Neurosurgery, Rady Children’s Hospital, San Diego, CA 92123, USA; 6 Department of Neurosurgery, University of California San Diego, La Jolla, CA 92093, USA; 7 Department of Surgery, Section of Otolaryngology, University of Chicago Medicine, Chicago, IL 60637, USA; roxbury@uchicago.edu; 8 Division of Pediatric Otolaryngology, Rady Children’s Hospital, San Diego, CA 92123, USA; 9 Department of Otolaryngology-Head and Neck Surgery, University of California San Diego, La Jolla, CA 92093, USA

**Keywords:** pediatric, endoscopic, endonasal, skull base surgery, reconstruction, technical considerations, multidisciplinary team

## Abstract

Originally pioneered in adults, endoscopic endonasal approaches for skull base pathology are being increasingly applied as a minimally invasive alternative for young children. Intrinsic anatomic differences between these patient populations have sparked discussions on the feasibility, safety, and efficacy of these techniques in pediatric patients. This work aims to serve as a primer for clinicians engaged in the rapidly evolving field of pediatric endoscopic skull base surgery. A succinct overview of relevant embryology, sinonasal anatomy, and diagnostic workup is presented to emphasize key differences and unique technical considerations. Additional discussions regarding select skull base lesions, reconstructive paradigms, potential surgical complications, and postoperative care are also highlighted in the setting of multidisciplinary teams.

## 1. Introduction

The adoption of endoscopic endonasal approaches, initially pioneered in adults for managing skull base pathology, has sparked debate regarding technical feasibility and surgical safety in young children. Inherent anatomic differences between these patient populations form the focal point of these discussions. Due to intricate complexities involving perioperative management as well as disease surveillance, management of pediatric patients with skull base disorders requires a multidisciplinary approach [1,2].

This work aims to serve as a primer for clinicians engaged in the rapidly evolving field of pediatric endoscopic skull base surgery. First, an overview of sinonasal embryology and pertinent anatomic differences is presented to elucidate unique challenges posed by the pediatric cranial base. Subsequently, the essential components of a comprehensive diagnostic workup and review of select pediatric skull base pathologies will also be highlighted. Finally, unique technical considerations, reconstructive approaches, potential surgical complications, and postoperative care will be explored in the setting of multidisciplinary teams.

## 2. Sinonasal Embryology

When considering anatomic variations with respect to age, two crucial facts should be noted. First, skull base development is a gradual, age-dependent process that significantly alters endoscopic endonasal corridors. Second, skull base lesions can markedly influence expected developmental and age-dependent growth patterns [3].

With respect to the paranasal sinuses, the maxillary and ethmoid sinuses begin their development at approximately the 10th week of gestation and are the only paranasal sinuses present at birth [4]. The maxillary sinus exhibits a biphasic postnatal growth pattern, reaching its final size by approximately 18 years of age. Ethmoid sinuses, being the first to develop in utero, exhibit the most advanced state at birth. Many of the cells are fully developed, though their size continues to mature over time. Originating as evaginations from the nasal wall, the anterior cells emerge initially from the middle meatus, followed by posterior cells from the superior meatus. The final size is typically attained by 12 years of age [4].

The frontal sinus initiates development as an upward extension from the frontal recess around the 16th week of gestation. It penetrates the frontal bone by 5 years of age and attains complete size during puberty. Paired evaginations from the sphenoethmoidal recess give rise to the sphenoid sinus at approximately the 4th week of gestation, and the pneumatization pattern significantly influences endoscopic endonasal approaches [4].

### 2.1. Nasal and Pyriform Apertures

The nasal apertures, or nostrils, represent the initial point that may impede endonasal access in the small pediatric nose. Radioanatomic studies indicate that the mean nasal aperture for patients aged 2–4 years is 6.7 mm, increasing to 9.3 mm by adolescence [3,4].

The pyriform aperture poses the second restriction point for skull base surgeons. Previous radioanatomic studies report a mean pyriform aperture diameter of 15 mm at birth, gradually increasing to 18 mm by age 2 years and exceeding 20 mm by age 5 years [5,6,7]. While these measurements may seem trivial, their clinical significance lies in the nearly doubled cross-sectional area of the aperture, transitioning from 15 mm to 20 mm in diameter [8]. Adapting to extremely young patients who require endonasal surgery may involve reducing endoscope size to a 3 mm lens, transitioning to three-handed approaches, and incorporating otologic micro-instruments into the pediatric skull base surgeon’s armamentarium.

### 2.2. Sphenoid Sinus Pneumatization

**Figure 1:**
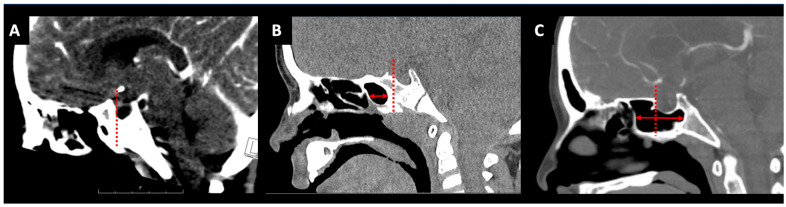
Sphenoid sinus pneumatization patterns. Conchal pattern (**A**), presellar pattern (**B**), and postsellar pattern (**C**). Red arrows show the pneumatized sphenoid sinus; the dashed line shows the tuberculum sellae plane.

Sphenoid sinus pneumatization patterns (Figure 1) play a crucial role in planning and executing safe and effective pediatric skull base surgery. In patients under 4 years of age, a conchal pneumatization pattern is most common. Pneumatization typically initiates around ages 2 to 4 and is completed by puberty, resulting in a fourfold increase in sphenoid sinus volume and a 50% expansion of the sella from its original size [9]. This process usually starts anteromedially near the sphenoid ostium, progressing posterolaterally and superiorly towards the planum sphenoidale and sella turcica. After approximately 10 years of age, it shifts towards a more posteroinferior direction, extending to the clival recess [8]. Consequently, the majority of pediatric patients do not exhibit dorsal pneumatization of the sphenoid sinus towards the clivus, thus obscuring the posterior margin of the sella.

Although previous reports suggest the degree of sphenoid sinus pneumatization may not directly impact outcomes, limited pneumatization can influence the extent of bony exposure required during surgery, consequently impacting operative time [8]. Preoperative imaging, including fine-cut computed tomography (CT) angiography and magnetic resonance imaging (MRI) scans for detailed bone, vascular, and soft tissue/tumor identification, along with a comprehensive review of key anatomic landmarks and intraoperative surgical navigation with periodic confirmation of neurovascular structures, may prove beneficial. These measures can enhance the safety and efficiency of bony drilling, ultimately reducing total surgical time for young patients.

### 2.3. Intercarotid Distance

While previous radiographic studies suggest that intercarotid distances within the clivus remain stable throughout life, variations may occur more superiorly within the cavernous sinus in patients under 4 years of age [4,9]. This becomes crucial when planning sellar, suprasellar, or transclival approaches in patients younger than 2 years of age, as an intercarotid distance below 10 mm may be restrictive for middle and posterior cranial fossa lesions [10]. Although this becomes less of a concern with anterior cranial fossa approaches in adolescents, assessing intercarotid distance in all patients remains essential. Neurovascular elements in this population are often thinner and more fragile when compared to adults, and congenital malformations may further alter these measurements [11].

## 3. Diagnostics

### General Workup

A comprehensive clinical history and thorough physical examination that includes nasal endoscopy and cranial nerve examination are fundamental for evaluating the majority of skull base pathologies. Nasal endoscopy is particularly crucial to assess sinonasal anatomy, evaluate macroscopic tumor characteristics, evaluate for underlying sinonasal infection, and determine viable reconstructive options. The decision to perform a biopsy should be made on a case-by-case basis. While biopsies are crucial for establishing a histopathological diagnosis for certain lesions, such as malignancies, they are contraindicated for vascular tumors or encephaloceles.

The preoperative imaging approach for skull base pathologies often includes a contrasted MRI and CT scans. MRI scans provide excellent soft tissue and tumor information. While MRI scans have no radiation exposure, it is important to note that these scans often require general anesthesia or sedation in infants and young children. Special MRI pituitary protocols that utilize thin 1–3 mm coronal slices through the sella turcica are preferred to evaluate for sellar lesions in relation to neurovascular structures [12]. CT scans provide complete information on paranasal sinus pneumatization and skull base configuration with the added benefit of utilization for intraoperative surgical navigation [4].

CT angiography plays a crucial role in delineating vascular pathologies and assessing the feasibility of preoperative embolization to minimize intraoperative blood loss. Importantly, angiography should not only be confined solely to vascular pathologies; it is also valuable for preoperative evaluation of intercarotid distance [9]. Comprehensive radiographic imaging, in general, is pivotal in informing surgical planning and potential reconstructive options. These deliberations are ideally conducted within a multidisciplinary conference involving members of a pediatric skull base team.

A comprehensive endocrine panel is crucial for discerning sellar lesions [9]. It facilitates prompt identification and correction of potential life-threatening hormonal imbalances while establishing the preoperative baseline function of the hypothalamic-pituitary axis. This panel includes, but is not limited to, levels of prolactin with dilution (PRL) (dilutions are necessary to avoid potential hook effect) [13], luteinizing hormone (LH), follicle-stimulating hormone (FSH), testosterone, insulin-like growth factor 1 (IGF-1), growth hormone (GH), thyroid-stimulating hormone (TSH), free triiodothyronine (T3), thyroxine (T4), cortisol, adrenocorticotropic hormone (ACTH), glycated hemoglobin (HbA1C), and alpha subunit.

Routine preoperative laboratory testing, which encompasses a complete blood count, basic metabolic panel, coagulation studies, and blood grouping with crossmatching, plays a crucial role in establishing a preoperative baseline. This process ensures the optimization of the patient’s health before major surgery and identifies individuals at high risk of intraoperative blood loss.

Ophthalmologic evaluation, including funduscopic examination and visual field testing, is crucial to establish preoperative baseline function and identify preoperative deficits, including papilledema, optic nerve pallor, extraocular muscle dysfunction, and visual field cuts [9]. Following surgery, repeat ocular examination may be required to evaluate for visual changes following tumor resection.

Tumor marker testing, encompassing alpha-fetoprotein (AFP) (with consideration for age-dependent values) [14] and beta-human chorionic gonadotropin (beta-hCG) in both blood and cerebrospinal fluid (CSF), proves invaluable in diagnosing nongerminomatous germ cell tumors. These tumors, which frequently resemble other sellar masses on imaging, warrant timely initiation of chemoradiation, potentially obviating the need for surgical biopsy [15].

In cases of hydrocephalus, careful consideration should be given to the need for CSF diversion, whether through an external ventricular drain or a ventriculoperitoneal shunt. Any mass-occupying lesion that obstructs the normal pathways of CSF flow can progress to cause obstructive hydrocephalus [9]. Considerations of the presence or impending presence of hydrocephalus should also be actively assessed.

## 4. Pediatric Skull Base Pathology (Figure 2)

### 4.1. Anterior Cranial Fossa

Anterior cranial fossa lesions pose unique surgical challenges, particularly in children, given the proximity to the frontal sinus with variable pneumatization patterns and central olfactory system.

**Figure 2:**
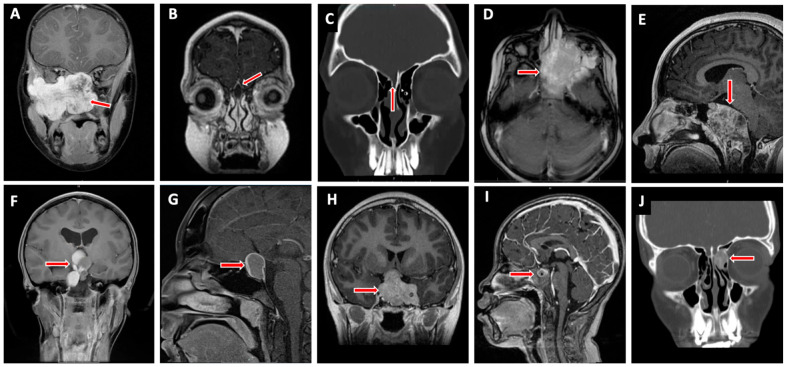
Commonly encountered pediatric skull base lesions. Juvenile nasopharyngeal angiofibroma (**A**), intracranial dermoid cyst (**B**), intranasal encephalocele (**C**), rhabdomyosarcoma (**D**), chordoma (**E**), craniopharyngioma (**F**), Rathke’s cleft cyst (**G**), pituitary adenoma (**H**), germ cell tumor (**I**), and fibrous dysplasia (**J**). Red arrow indicates pathology.

#### 4.1.1. Nasal Dermoid Cyst

Nasal dermoids are rare congenital lesions with an estimated incidence of 1:20,000–40,000 births that result from a failure of the dural diverticulum to separate from overlying skin through the foramen cecum [4,16,17]. These lesions account for approximately 10% of all head and neck dermoids and 60% of midline nasal masses [4,18]. This persistent connection between dura and dermis can be filled with hair and epithelial debris, presenting as a firm cystic mass, sinus, or fistula over the nasal dorsum with an associated midline pit [16,18,19,20]. Depending on the degree of dural connection, superimposed infection can spread intracranially, resulting in meningitis or cerebral abscess formation. Naturally, it is important to determine the degree of dural extension before any type of surgical intervention, which can be seen in 5–45% of lesions [16,21,22]. CT or MRI is recommended to evaluate for intracranial dermoid cyst extension, but there is a debate over which modality, not if both, should be obtained preoperatively [23]. CT may be fast and highlight osseous structures, but there is significant concern for radiation exposure, especially for young children. MRIs negate this concern, but often require sedation and long acquisition times [23]. According to a recent single-institution study comparing the accuracy of CT and MRI in predicting intracranial extension for frontonasal dermoids, the authors demonstrated that the sensitivity and specificity of CT were 87.5% and 97.4%, respectively, whereas MRI was 60.0% and 97.8%, respectively. The authors concluded that a single modality should be chosen given comparable accuracy. MRI was generally preferred in their analysis [23]. In the setting of nasal dermoid cysts, CT may demonstrate bifid crista galli, which may be suggestive of a skull base defect [24]. MRI often reveals a heterogeneous mass on T1-weighted sequences, whereas T2-weighted sequences may show a well-circumscribed, moderately hyperintense lesion [25]. Moreover, a hallmark of nasal dermoids is that they are diffusion-restricted akin to cholesteatoma, which may help differentiate them from vascular lesions or other low-grade tumors.

#### 4.1.2. Encephalocele

Encephaloceles are aberrant outpouchings of intracranial contents into sinonasal spaces through either congenital or acquired skull base defects. Congenital encephaloceles have an estimated incidence of 0.2 per 1000 live births [26]. Described by their contents (i.e., meningocele for outpouching of meninges and CSF versus meningoencephalocele for outpouching of meninges, CSF, and brain tissue), these lesions may be identified incidentally on imaging or may present with nasal obstruction, CSF rhinorrhea, or meningitis [4]. Clinical examination and nasal endoscopy may reveal a soft and compressible sinonasal mass that may enlarge with crying or with jugular vein compression (Furstenberg sign) [18,20]. These lesions can be further divided into sincipital and basal encephaloceles. Sincipital encephaloceles have a facial component and can be described by their anatomic location (i.e., frontonasal, -ethmoidal, or -orbital). Basal encephaloceles can also be described by their location and tend to present with airway obstruction [18,27]. Skull base defects can be lateral to the foramen cecum with variable extension into the ethmoid roof and cribriform, or isolated to the ethmoid roof with the appearance of low-lying funnel-shaped anterior skull base defects on CT imaging [18,28].

MRI will define prolapsed intracranial components along with its effect on adjacent neurovascular structures. Typical findings include cerebral tissue protruding towards the defect with elongation of adjacent ventricles [29]. CT imaging will demonstrate associated skull base osseous defects in most cases. Careful consideration should be given to potential vascular entrapment within the encephalocele, which may lead to challenging hemorrhage management and large subclinical infarcts, especially when affecting the frontal lobes.

#### 4.1.3. Juvenile Nasopharyngeal Angiofibroma

Juvenile nasopharyngeal angiofibromas (JNAs) are rare, benign but locally invasive, highly vascular tumors that arise in the vicinity of the sphenopalatine foramen [4,18,27]. JNAs present exclusively in adolescent males with severe recurrent epistaxis and nasal obstruction due to the androgenic hormone required for their growth [20,30]. Although JNAs are generally localized to the nasopharynx, up to 36% of cases can extend intracranially [18,27]. A subset of intracranial pathology can be quite extensive, involving the cavernous sinus and internal carotid arteries, and may present with visual changes or other central nervous system (CNS) symptoms [4,18]. CT imaging often demonstrates anterior bowing of the posterior wall of the maxillary sinus (Holman–Miller sign) secondary to pterygomaxillary tumor extension. On MRI, lesions are often heterogeneous with mixed hyper- and isointense appearance in both T1-weighted and T2-weighted sequences, and enhance avidly with contrast [31]. Preoperative biopsy is not recommended due to risk of hemorrhage [18,20,32]. Preoperative embolization by an experienced neurointerventional team is very helpful in reducing the amount of expectant intraoperative blood loss during tumor resection. Pre-embolization angiography should define anastomoses relevant to critical anatomic structures and for reconstructive considerations before sacrifice. Of note, the University of Pittsburgh Medical Center (UPMC) staging system considers two important prognostic factors: cranial base extension route and vascularity. It offers superior prediction of immediate morbidity and tumor recurrence compared to other tumor staging systems [33].

#### 4.1.4. Esthesioneuroblastoma

Also known as an olfactory neuroblastoma, an esthesioneuroblastoma is a malignant tumor of neuroectodermal origin thought to originate from the basal layer of the olfactory epithelium located in the superior third of the nasal septum, cribriform plate, and superior turbinates [27,34]. This is a rare cancer with an estimated annual incidence of 0.4 per 1,000,000, accounting for 6% of sinonasal malignancies [34]. Presenting symptoms are related to the contiguous spread of disease through the cribriform plate involving the anterior cranial fossa, orbit, and brain; namely, anosmia, recurrent epistaxis, nasal obstruction, orbital proptosis, restricted extraocular eye movements, and visual impairments. Commonly accepted tumor staging has been described by Kadish et al. [35]. Five-year survival rates are estimated to be between 40% to 80% and are closely associated with the pathological grade and tumor staging [27,36]. On CT imaging, an esthesioneuroblastoma appears as a nonspecific soft tissue mass in the nasal vault with possible adjacent bony remodeling or erosion. On MRI, these lesions are typically hypointense on T1-weighted sequences and intermediately hyperintense on T2-weighted imaging. Esthesioneuroblastomas enhance with contrast administration and are often associated with cystic lesions when there is intracranial extension [35].

#### 4.1.5. Rhabdomyosarcoma

Rhabdomyosarcomas are one of the most common malignant pediatric soft tissue tumors, although they remain rare, with an estimated incidence of little over 100 cases per year in the United States [37,38,39]. Several hereditary disorders, such as Li–Fraumeni syndrome, neurofibromatosis type 1, Gardner syndrome, hereditary hemochromatosis, and Werner syndrome, have been associated with rhabdomyosarcoma; however, no clear association has been identified [38]. Sinonasal rhabdomyosarcomas, especially the alveolar subtype, generally exhibit poor outcomes. However, those under 18 years old and with embryonal or botryoid subtypes may have a relatively better prognosis [20,40]. Patients generally present with a painless mass with variable symptoms based on size and location. Tumors arising in the submucosal sinonasal tract and anterior skull base often present with nasal congestion, recurrent epistaxis, or visual disturbances [38]. Although diagnosis is guided by biopsy, CT and MRI can help delineate the extent of bony involvement, intracranial extension, and regional metastasis. The vast majority have homogeneous enhancement on contrasted MRI scans and will be best visualized on T1-weighted sequences. Asymmetrical enlargement of the cavernous sinus with abnormal enhancement in skull base foramina may indicate intracranial tumor extension along trigeminal nerve branches. Radiographic evaluation should focus on the obliteration of normal fat planes and abnormal contrast enhancement when compared to the non-involved side [41]. The role of upfront surgery beyond biopsy is debatable, given its exceptional response to chemoradiation [42].

#### 4.1.6. Fibrous Dysplasia

Fibrous dysplasia is a rare localized disorder characterized by abnormal proliferation of fibrous–osseous tissue replacing normal bone [43]. There are three disease forms: monostotic, polyostotic, and McCune–Albright syndrome. Importantly, the monostotic variant may stabilize after puberty, while the polyostotic form may persist into adulthood [44]. Approximately 70–80% of fibrous dysplasia presents as a monostotic form affecting the ribs and craniofacial skeleton, and is considered to be the mildest form, generally presenting between ages 20–30 years [18,45]. Polyostotic fibrous dysplasia (20–30%) is typically diagnosed earlier in childhood and presents with more severe skeletal and craniofacial involvement [45]. McCune–Albright syndrome is the most severe form of the disorder, affecting approximately 3% of cases [45]. This syndrome is more common in females and is associated with short stature, café au lait spots, and endocrinopathies [18,45]. Given the expansile nature of this disease, skull base involvement can result in progressive neurovascular impingement leading to cranial neuropathy and functional impairment. Common anatomic locations involved include the ethmoid, sphenoid, and frontal bones, with orbital involvement in up to 43% of cases [43]. Fibrous dysplasia can also present as atypical facial pain/headache, sinusitis, proptosis/diplopia/vision changes, and facial/skull enlargement [43]. On CT imaging, fibrous dysplasia may present as sclerotic or compact lesions with characteristic ground-glass appearance (50%), lytic lesions with an eggshell appearance (15%), or mixed/pseudopagetoid lesions (35%) [18]. Special attention should be paid to localizing areas of nerve impingement. Tortuosity of large extracranial vessels may lead to stenosis and/or pseudoaneurysm formation and warrant detailed assessment and monitoring. Current indications for surgery include optic nerve impingement or cranial neuropathy. Cosmetic remodeling remains quite controversial [32]. When considering fibrous dysplasia as a diagnosis, one should consider ossifying fibroma and osteoma as differentials [46].

### 4.2. Middle Cranial Fossa

The most common middle cranial fossa tumors in children are related to the pituitary gland and craniopharyngeal duct [47]. These sellar lesions can cause mass effect on the optic nerves and chiasm, resulting in progressive visual disturbances [9,27]. Classically, central middle cranial fossa lesions in children present with endocrinologic derangements such as panhypopituitarism, precocious puberty, secondary amenorrhea, and diabetes insipidus [27]. These lesions may present with bitemporal hemianopsia due to mass effect on the optic chiasm or as diabetes insipidus due to disruption of the hypothalamic–infundibular–pituitary axis [9,48].

#### 4.2.1. Rathke’s Cleft Cyst

Rathke’s (pars intermedia) cleft cysts (RCCs) are benign cystic lesions thought to arise from remnants of the ectopic embryonic Rathke’s pouch within the pituitary gland. Generally, these lesions are slow-growing and thus commonly asymptomatic in children, but may exert mass effect with continued growth in the sella and possible extension into the suprasellar region. Often found incidentally for chief complaints including recurrent headaches, visual changes, or hormonal derangements, these lesions must be differentiated from craniopharyngioma or pituitary adenoma [4,20,49]. On T1-weighted sequences, RCCs can appear either hyperintense or hypointense on MRI, depending on the degree of proteinaceous contents and inflammation. On T2-weighted sequences, these lesions generally appear hyperintense. Moreover, RCCs are non-enhancing and may have intracystic nodules in over 75% of cases that are hyperintense on T1-weighted and hypointense on T2-weighted sequences [50].

#### 4.2.2. Craniopharyngioma

Craniopharyngiomas are one of the most common CNS neoplasms in children and are thought to arise from ectodermal remnants of the Rathke’s pouch or residual epithelium of the embryonal hypophysis and tuber cinereum or components of the third ventricular floor. This is a challenging disease owing to the tumor’s large dimensions, location, and refractory nature. There are two major histologic subtypes. In children, cystic adamantinomatous tumors are most prevalent when compared to the papillary form seen in adults [51]. Younger children tend to present with signs and symptoms of obstructive hydrocephalus that include headache, nausea, vomiting, lethargy, or more subtle cognitive changes from mass effect, whereas adolescents and young adults can present with growth restriction, delayed puberty, or visual disturbances [20,27,52]. On imaging, craniopharyngiomas may appear as a complex, polycystic, and expansile mass with mixed density and intensity on CT and MRI, respectively [51,52]. However, the presence of calcification in a complex sellar and suprasellar tumor is suggestive of craniopharyngioma and can help differentiate it from pituitary adenoma and other rare suprasellar neoplasms [51,52]. Proton MR spectroscopy may be revealing, given the unique signature characterized by a single, dominant lipid–lactate peak seen in craniopharyngioma when compared to high choline-to-N-acetylaspartate ratio observed in glioma and the bland signature of pituitary adenomas [53]. An assessment should be made as to whether gross total vs. subtotal resection should be attempted. Given the anatomic location, craniopharyngiomas also require special attention to the hypothalamus and optic chiasm [8,54]. Should involvement of the hypothalamus be suspected, subtotal resection may be preferable with a plan for adjuvant radiation therapy [4]. Recent advancements in targeting molecular changes (BRAF V600E) in papillary craniopharyngiomas have led to promising adjuvant therapies for this difficult-to-treat pathology [55].

#### 4.2.3. Pituitary Adenoma

Pituitary adenomas are rare and represent 3% of all intracranial neoplasms [12,56]. Categorized by a secretory state, functional tumors comprise the vast majority of pituitary adenomas in children, in contrast to the adult population [12]. Perry et al. conducted a review of 37 surgical series published since 1970 (1284 patients) and demonstrated ACTH-secreting tumors were the most common (43%) functional pituitary adenomas in children, followed by PRL-secreting (37%) and GH-secreting (12%) [56]. Presenting symptoms depend on the age, location, size, and functional state of the pituitary adenoma. Although rare, GH-secreting adenomas may present as gigantism if present before the fusion of long bone growth plates or as acromegaly if presentation is later [12,56]. On MRI, pituitary adenomas are usually hypointense on T1-weighted imaging with delayed contrast enhancement [48]. Prolactinomas represent a unique subset of pituitary tumors, as they are sensitive to dopamine agonists.

#### 4.2.4. Germ Cell Tumor (GCT)

Germ cell tumors (germinoma, nongerminomatous germ cell tumor (yolk sac, endometrial sinus tumors, embryonal carcinoma, and choriocarcinoma), and teratoma) represent 3–11% of pediatric intracranial neoplasms, with up to one third occurring in the sellar/suprasellar region [9]. On MRI, GCTs are often isointense with a strong enhancement of solid components with gadolinium. Cystic components will appear hyperintense on T2-weighted sequences without contrast enhancement [48]. Germ cell tumors should initiate a systemic workup, as well as an assessment of the pineal region for metastases.

Teratomas are a rare type of germ cell tumor with a heterogeneous mixture of differentiated tissues from all three embryonic layers. Head and neck manifestation of a teratoma is even more rare with highly variable presentation [57]. They may present as a small pedicled mass to a large mass extending intracranial from oral and nasopharyngeal cavities. Teratomas can be associated with other congenital anomalies and commonly present as an airway obstruction in the neonatal period [18]. CT and MRI may show a heterogeneous, well-circumscribed tumor with a mixture of cystic and solid components including aberrant anatomic structures (teeth, hair, etc.) [57].

#### 4.2.5. Langerhans Cell Histiocytosis/Eosinophilic Granuloma

Langerhans cell histiocytosis is a rare disease characterized by the spectrum of aberrant proliferation and accumulation of antigen-presenting immune cells called Langerhans cells around the body [58]. Multicentric disease can affect the pituitary infundibulum, giving it a thickened appearance on MRI, with associated endocrinopathies such as diabetes insipidus [59,60]. The mildest form of Langerhans cell histiocytosis is eosinophilic granuloma, which may present as an osteolytic lesion of the calvarium and skull base [61,62]. On CT, eosinophilic granuloma may appear as a soft tissue mass with associated bony destruction. On MRI, the lesion may appear hypointense to isointense with a diffuse contrast enhancement on T1-weighted sequences and may appear hyperintense on T2-weighted sequences [61]. On histologic analysis, there may be characteristic Birbeck granules, which may aid in diagnosis. These lesions are typically sensitive to corticosteroids and directed chemotherapy. Biopsy is reserved for rare situations, given the high risk to the pituitary infundibulum.

### 4.3. Posterior Cranial Fossa

Endoscopic endonasal approaches to the posterior fossa have expanded surgical options to treat a variety of diseases within this region [63]. Presenting symptoms may vary from dysphagia, nasal obstruction, visual disturbances, headaches, neck pain, and cranial neuropathies based on anatomic region ranging from dorsum sella, clivus, jugular tubercle, and occipital condyles affecting the brainstem, atlantooccipital joint, and lower cranial nerves [27,63]. Advancements in the reconstructive paradigm with modern rhinologic techniques have alleviated historical concerns that previously limited this approach.

#### 4.3.1. Chordoma/Benign Notochord Cell Tumor

Chordoma is a classically slow-growing, locally aggressive, and indolent neoplasm arising from embryologic notochordal remnants with a reported yearly incidence of 0.3–0.8 per million worldwide [64]. Although chordoma can arise anywhere along the axial skeleton, pediatric chordomas affect the clivus more commonly. Patients may present with pain, visual disturbances, particularly abducens nerve palsy, nasal obstruction, or failure to thrive [20,65]. Diagnosis can be confirmed by biopsy, which may show one of three histologic subtypes: classical (i.e., physaliferous), chondroid/mesenchymal, or dedifferentiated. The first two subtypes portend a better long-term prognosis [4,66]. On CT, chordoma may appear as a well-defined expansile clival mass with associated osteolytic lesions and adjacent soft tissue involvement. On T1-weighted sequences, there may be focal hyperintensity within hypointense lesions representing focal hemorrhage or mucinous content on MRI. These tumors are classically hyperintense on T2-weighted sequences [66]. Contrast enhancement is variable.

Benign notochord cell tumor (previously known as ecchordosis physaliphora) is a rare, benign notochordal remnant often mistaken for chordoma. A recent systematic review of the English literature identified only 60 cases of symptomatic CNS ecchordosis physaliphora, 5 of which were in pediatric patients, highlighting their rarity [67]. Due to their histologic and radiographic similarity to chordoma, these lesions pose challenging dilemmas in diagnosis and management. However, important imaging characteristics that may help distinguish ecchordosis physaliphora from chordoma are a well-circumscribed appearance of dorsal and retroclival lesions, absence of bony erosions, and the presence of pedicles or stalks that are hypointense on T2-weighted MRI [34]. A careful review of previous imaging studies may reveal the stable nature of these lesions, helping to distinguish them from chordomas [68].

#### 4.3.2. Chondrosarcoma

Intracranial chondrosarcomas are rare tumors thought to arise from nests of endochondral cartilage in the petroclival fissure [69,70]. As such, these tumors affect areas of the skull base that mature predominantly by endochondral ossification. Clival chondrosarcoma can present similarly to chordoma based on involved anatomic location. Although most chondrosarcomas are sporadic, they can be seen in patients with Ollier’s disease, Maffucci syndrome, Paget’s disease, and osteochondroma [71]. Histologically, chondrosarcomas can be subtyped into conventional, mesenchymal, clear-cell, and dedifferentiated chondrosarcoma, with conventional and mesenchymal tumors being more commonly described in the literature [66,71]. Establishing a definitive preoperative diagnosis may be challenging, due to their resemblance with chordoma [66]. However, chondrosarcomas tend to be more lateral than chordomas and can be seen centered around the petroclival synchondrosis [72].

#### 4.3.3. Other Skull Base Lesions

Although several pediatric skull base pathologies have been discussed in this article, this is certainly not an exhaustive list of all potential pathologies described in the medical literature. Although rare, it is important to keep in mind a broad spectrum of differential diagnoses, such as meningeal tuberculosis or other infectious etiologies, diaphragma sellae meningioma, hemangioma, and numerous granulomatous and fibro-osseous diseases such as giant-cell reparative granuloma and osteoma when considering pediatric skull base lesions [27,46,48,72,73,74].

## 5. Multidisciplinary Treatment Planning

Collaborating with adult skull base teams may enhance the pediatric treatment experience, increasing exposure to complex surgical planning and radiologic nuances. However, a pediatric-centered skull base team can often independently tailor treatment plans to meet the comprehensive psychosocial, rehabilitative, and developmental needs of children.

**Figure 3:**
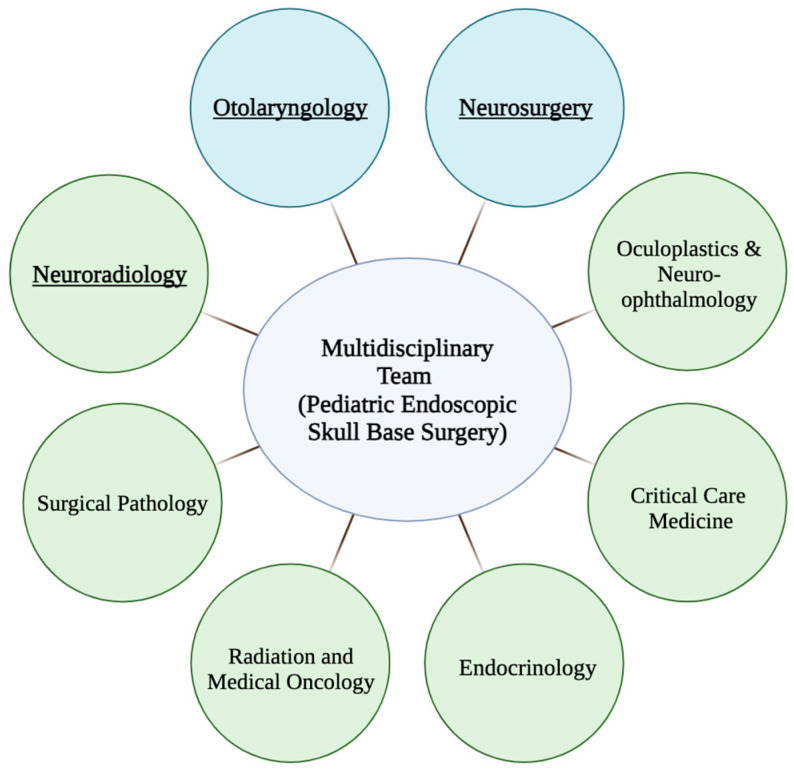
Components of a successful multidisciplinary team for pediatric endoscopic skull base surgery.

The principal surgical team engaged in skull base procedures depends on institutional protocols, patient demographic profiles, and the specific pathological conditions under consideration. Typically, core team members include neurosurgeons, neuroradiologists, and otolaryngologists (Figure 3). Expertise in neuroradiology is paramount for accurate imaging interpretation, facilitating the establishment of a precise differential diagnosis, and tumor extent assessment [75]. Additionally, it is imperative for the multidisciplinary team to meticulously formulate and assess surgical strategies, ideally incorporating virtual endoscopic techniques, reconstruction protocols, and surgical planning methodologies [76].

Collaborative tumor board discussions further enhance patient care for skull base diseases. These include the medical disciplines of endocrinology, oculoplastics, ophthalmology, pathology, medical oncology, radiation oncology, endocrinology, and critical care (Figure 2) [75,77]. Accurate pathologic diagnosis is crucial due to varying natural histories and treatment options for differing conditions. Intraoperative neuromonitoring using triggered electromyography is increasingly integrated into endoscopic endonasal surgeries due to its crucial role in identifying cranial nerves and averting neurological injuries [78]. Additionally, high-grade malignancies may require adjunctive therapies, especially in an induction/neoadjuvant context, to improve locoregional control, reduce local therapy-related morbidity, and lower the risk of distant metastasis [64,77].

Furthermore, collaboration between academic institutions and centers of excellence is vital for advancing pediatric skull base treatments, especially in low-volume settings. The scarcity of evidence-based standard of care underscores this necessity. Without such centers and patient care standardization, treatment variations may be influenced by individual physicians’ training and preferences. Multi-institutional collaboration aids in disseminating current techniques, boosting the technical proficiency of pediatric surgical teams [2].

Advancements in radiographic imaging have aided preoperative assessment of important neurovascular structures in planning the optimal operative corridor. Intraoperative image guidance has become an important tool for identifying important anatomic landmarks, especially when drilling a poorly pneumatized pediatric skull base [9]. However, the importance of the skull base surgeon’s ability to maneuver the complex three-dimensional (3-D) space cannot be understated in executing a successful endoscopic skull base tumor resection [79].

Three-dimensional-printed models have been shown to improve surgical outcomes by assisting in reconstruction, reducing operative time, minimizing blood loss, and lowering the incidence of surgical complications. The use of patient-specific 3-D models has been shown to be more beneficial than two-dimensional (2-D) images when consenting patients or discussing preoperative plans with trainees. With familiarity and efficient workflow, Langdon et al. showed that 3-D-printed models can be swiftly designed and accurately fabricated in approximately 24 h [80]. The ability to rapidly deploy and incorporate patient-specific 3-D models in skull base surgery certainly has a promising future in surgical safety, patient education, and training.

## 6. Endoscopic Endonasal Approach

One of the key challenges of pediatric endoscopic skull base surgery is sufficient exposure. As previously discussed, the nasal apertures often limit adequate instrumentation access. Even so, a 4.0 mm, 0-degree nasal endoscope is preferable when feasible, since it allows for optimal visualization and illumination of the operative field.

In cases of intracranial dissection, perioperative antibiotics with CSF penetration should be given. Traditionally, weight-based ceftriaxone can be given or vancomycin + aztreonam for patients with a documented severe penicillin allergy. The nasal cavity should first be decongested with oxymetazoline. Afterwards, the inferior turbinates should be lateralized to improve endonasal access in the small pediatric nose. Total sphenoethmoidectomies will allow for complete visualization of the anterior cranial base. Rastatter et al. described the selective removal of the right middle turbinate to allow for nasal endoscope access [4]. Bilateral middle turbinate resection should be strongly considered if these maneuvers do not improve access, particularly for tumor resection [8].

These considerations are important to allow for a four-handed, two-surgeon technique. A wide sphenoidotomy should always be performed to allow for complete access to the planum and tuberculum sella [80]. Reconstructive options should be always assessed before performing sphenoidotomies. Often, a nasoseptal flap is raised upfront if a CSF leak is anticipated following tumor resection or bilateral rescue flaps are raised to preserve both posterior septal artery pedicles. As discussed previously, sphenoid pneumatization does not start until 2–4 years of age. The sphenoid sinus is 90% pneumatized by 6–7 years [81]. Patients younger than this may require drilling to allow for adequate sellar access. This is not an absolute contraindication to surgery, but places the patient at greater risk of complications [82]. Pneumatization of the sphenoid sinus is typically complete by age 10 [5,83]. However, pediatric sphenoid pneumatization has not been noted to prevent gross total resection [84]. If surgery is performed on a poorly pneumatized sphenoid sinus, blood loss should be expected due to the vascular nature of marrow and cancellous bone. This bone can be removed by a diamond burr to minimize blood loss until the planum sphenoidale is reached [85]. Intraoperative Doppler should be utilized to assess the location of the internal carotid arteries during drilling [85,86]. Furthermore, surgical navigation is critical in this role, as intraoperative landmarks are very limited in these cases.

### Technical Considerations

Reduced pediatric total blood volume and robust sinonasal vascularity may lead to substantial blood loss over time if meticulous hemostatic control is not maintained, even with low-flow venous bleeding and mucosal oozing. Employing a combination of techniques such as cauterization, nasal pledgets, warm saline irrigation, hemostatic agents, selective embolization (when indicated), or arterial ligation can significantly reduce intraoperative blood loss. Adequate preoperative planning and meticulous surgical technique play crucial roles in minimizing the risk of iatrogenic vascular injury [4].

While recent publications have examined midfacial growth in children undergoing expanded endonasal approaches and suggest no significant impact [9], it remains crucial to be mindful of the location of major sinonasal and skull base growth centers. The potential disturbance of these regions, particularly before their fusion (e.g., spheno-occipital synchondrosis, the primary axis for skull base growth), theoretically could have a negative influence on craniofacial development [4].

The anesthesia team should consider and address potential rapid drops in body temperature during surgery, a consequence of the higher body surface area-to-volume ratio in pediatric patients [87]. Consideration should also be given to coagulopathy in the setting of blood loss and avoiding hypotension in the setting of optic nerve compression. While intraoperative corticosteroids are a consideration, data are lacking on specific indications outside of documented adrenal insufficiency [88].

## 7. Skull Base Reconstruction

Pediatric postoperative CSF leak rates after skull base surgery are notably higher than adults [89]. This accentuates the need for a thoughtful approach towards skull base reconstruction. For small CSF leaks, dural substitute and free mucosal onlay grafts can be considered. For larger skull base defects, especially in the sella, multilayer closure with vascularized flaps should be considered [90]. Taken together, a reconstruction algorithm should consider underlying pathology, defect size, and CSF leak flow. A robust reconstruction model should include a combination of inlay and onlay grafts.

The nasoseptal flap (NSF) is the workhorse for skull base reconstruction. Multiple studies support NSF use in the pediatric population [91,92]. In children, it is important to measure expected NSF length. Shah et al. suggested that while NSF has adequate width for skull base defects in pediatric patients, it may fail to adequately provide the anterior–posterior length required for transcribriform and transclival defects [93].

There have further been concerns regarding the NSF’s impact on midface development in the pediatric population. However, maintaining normal mucosa over the contralateral septal lining has been hypothesized to mitigate these concerns as well as donor site reconstruction using a free mucosal graft from the resected middle turbinate [94,95].

Other rotational flaps that have been employed include the middle turbinate flap (MTF), lateral nasal wall flap [96], anterior ethmoid artery flap, and temporoparietal fascia flap (TPFF). For smaller defects, the MTF can be considered [97]. Based on the middle turbinate branch of the sphenopalatine artery, the mucosa of the middle turbinate can be elevated to cover defects of the fovea ethmoidalis, planum sphenoidale, or sella [97]. The mucosa will often maintain memory, which is a key limitation of this flap. The TPFF is a tunneled flap that can serve as an adjunct to the nasoseptal flap, with new techniques that have unrestricted access to the entirety of the skull base [98,99,100,101].

For posterior cranial fossa defects, a rhinopharyngeal (RP) flap should be considered. Based on the ascending pharyngeal artery, this flap is an inferiorly based pedicled flap that can be used in conjunction with a nasoseptal flap for adequate coverage of clival defects [102].

Other methods of reconstruction include autologous grafts. Fascia lata harvested from the upper leg has been described as part of a multilayer skull base reconstruction. Abdominal fat from a periumbilical incision can also buttress a skull base reconstruction. The latter minimizes the risk of pontine herniation when combined with a nasoseptal flap [103]. Additional adjuncts, including fibrin glue and dural sealants, can be applied to provide a watertight closure across a skull base reconstruction [11,104]. Finally, the bolster for skull base reconstruction can be either absorbable packing (i.e., gelatin sponge, oxidized regenerated cellulose, etc.) or nonabsorbable packing (i.e., surgical sponges).

Lumbar drain placement is controversial in the pediatric population. It has been suggested that lumbar drains should be placed in cases of high-flow intraoperative CSF leaks, decompression surgery, or revision cases [105,106,107].

## 8. Surgical Complications

Complications for pediatric skull base surgery are fortunately low. Most reported complications parallel adult skull base surgery. For craniopharyngioma and sellar lesions, this includes damage to surrounding neurovascular structures [51]. For craniopharyngiomas, great care must be taken to avoid hypothalamic injury [108,109]. Despite these considerations, diabetes insipidus will occur in up to 80% of patients postoperatively [52]. These patients will often require lifelong growth hormone and thyroid hormone supplementation. These patients should be counseled regarding these risks and the possibility of stalk sacrifice, as well as the need for close endocrinology management.

A further consideration is vision deterioration in patients with sellar and suprasellar lesions. Although many of these patients present with visual changes, surgery must avoid damage to the optic nerves and chiasm to minimize further visual compromise [110].

Vascular injury is an important consideration with skull base surgery [8]. In transcribriform approaches, great care must be taken to avoid injury to the anterior and posterior ethmoidal arteries, and often upfront vascular control and ligation with bipolar electrocautery is essential to avoid retraction of the vessels into the orbit. In the transplanum and sellar approaches, intraoperative Doppler can aid the surgeon in avoiding injury to the parasellar carotid arteries. In anticipation of these concerns, either the leg or abdomen should be prepped and draped to allow rapid access to a free rectus muscle or vastus lateralis muscle graft to help assist with tamponading an internal carotid injury. In cases of vascular compromise intraoperatively, following control with nasal packing, both CT angiography and/or digital subtraction angiography should be considered.

Cranial neuropathies should be considered when tumor resection involves the course of sensory nerves. Patients should be counseled on dry eyes and facial paresthesia owing to dissection around branches of the Vidian and trigeminal nerves, respectively [111,112].

## 9. Postoperative Care

Postoperatively, the patient should be monitored in the intensive care unit (ICU) for neurological checks, vascular checks, and monitoring of endocrinological stability [8]. Typical, ICU duration is 1.8–4.5 days following pediatric skull base surgery [113]. In the case of craniopharyngioma, diabetes insipidus is commonplace, and judicious fluid management is important [8]. There are no specific guidelines for radiographic imaging following surgery, but CT usually is performed postoperatively [114,115,116]. MRI should be considered within 24–48 h to evaluate the extent of resection, particularly in cases where surgery will be staged to address residual tumor (postoperative surgical changes may obscure interpretation of MR findings).

Other considerations specific to pediatrics include patient compliance. Persistent agitation, cough, and accidental nose blowing may compromise skull base repair. In these cases, consideration of patient sedation may be necessary [107,117].

Nasal saline sprays and irrigation are often not considered until after the first postoperative debridement. This is due to the limited ability of most young patients to elicit signs and symptoms concerning for CSF leak. Typically, nasal debridement occurs 1–2 weeks after surgery to ensure adequate healing of the skull base repair and confirmation of no occult CSF leak. Subsequent debridements will occur 4–6 weeks after surgery and then 3 months after surgery. Depending on the child’s overall functional status and age, consideration must be given to nasal debridement under general anesthesia if there are safety concerns noted regarding the ability of a patient to tolerate in-office nasal endoscopy and debridement.

Disease surveillance should be performed for all patients at regular intervals, 3–6 months, following surgery, particularly for patients undergoing adjuvant therapy (i.e., radiation). Surveillance nasal endoscopy ensures patients achieve baseline nasal function during the recovery process as well as monitoring for any long-term sinonasal morbidity.

## 10. Strengths and Limitations

This work provides a comprehensive guide for clinicians in pediatric endoscopic skull base surgery, bridging the gap between established practices and emerging trends. This report builds on previous efforts over the last decade [4,118], with a specific focus on modern management and multidisciplinary care of pediatric skull base patients.

Moreover, the discussion on endoscopic approaches extends beyond procedural techniques to encompass perioperative considerations, including antibiotic selection and anesthesia considerations, addressing practical challenges frequently encountered by surgeons. Contemporary reconstructive paradigms beyond the nasoseptal flap and ancillary surgical planning methods such as 3-D modeling are highlighted, showcasing late-breaking advances in this rapidly evolving field.

While recent advancements in medical technology and surgical experience have greatly expanded the horizons of safe and effective minimally invasive approaches to the pediatric cranial base, traditional open approaches maintain a role in pediatric skull base surgery, although they are not specifically covered in this focused review [119]. Finally, this report was assembled to specifically address the perioperative care of patients undergoing solely endoscopic skull base surgery. Given the relative rarity and significant heterogeneity of the clinical conditions covered in this state-of-the-art review, the ability to provide robust data-driven conclusions remains limited.

## 11. Conclusions

In children, the challenges of skull base surgery are paramount and include their small size, variable location of key anatomic landmarks due to craniofacial underdevelopment, and lack of outcome reporting of pediatric cases in the medical literature [120]. This underscores the critical role of pediatric multidisciplinary teams, given their diverse expertise and synergistic technical skills [76]. With expanding advances in optics and medical devices, pediatric skull base surgery has the potential to dramatically reduce the morbidity of surgical approaches. This accentuates the need for further concerted research efforts to establish best practices for treatment of this unique patient population.
